# Efficacy of an aluminium triformate mouthrinse during the maintenance phase in periodontal patients: a pilot double blind randomized placebo-controlled clinical trial

**DOI:** 10.1186/s12903-016-0214-z

**Published:** 2016-05-23

**Authors:** Adriano Azaripour, Jens Weusmann, Carl Eschig, Irene Schmidtmann, Cornelis J. F. Van Noorden, Brita Willershausen

**Affiliations:** Department of Operative Dentistry and Periodontology, University Medical Center, Johannes Gutenberg University Mainz, Augustusplatz 2, 55131 Mainz, Germany; Department of Cell Biology and Histology, Academic Medical Center, University of Amsterdam, Amsterdam, The Netherlands; Institute of Medical Biostatistics, Epidemiology and Informatics (IMBEI), Johannes Gutenberg University, Mainz, Germany

**Keywords:** Mouthrinse, Periodontal maintenance, Gingivitis, Dental plaque

## Abstract

**Background:**

The aim of this prospective placebo-controlled pilot study was to evaluate short-term effects of a mouthrinse containing aluminium triformate (ATF) on gingival inflammation and plaque formation in periodontal patients who are in the maintenance phase. ATF has styptic (astringent) and anti-inflammatory effects.

**Methods:**

Forty non-smoking periodontal patients with modified sulcus bleeding index (MSBI) ≥40 % were randomly divided into two groups. The participants received a masked mouthrinse (ATF or placebo) and were instructed with the rinsing protocol of 3 daily rinses during 30 s for 7 days. One blinded investigator (CE) performed all clinical examinations. The primary outcome was reduction in gingival inflammation as measured by MSBI. The secondary outcomes were reduction of the amount of plaque as measured by plaque index (PI) and approximal plaque index (API) and the occurrence of side effects. The patients were evaluated at the start and the end of the rinsing period, including the compliance of the patients.

**Results:**

MSBI was reduced in both groups compared to baseline, but the ATF group showed significantly more reduction in MSBI compared to the placebo group (ATF: 17.6 %, placebo: 7.6 %, *p* = 0.035). ATF and placebo had no effects on dental plaque. Patients reported ATF mouthrinse not to have side effects other than oral sensation, whereas compliance of the patients was good. Almost all patients in the ATF group reported reduction of gum bleeding after 1 week of rinsing with ATF.

**Conclusions:**

This short-term pilot clinical trial is a firm basis to design a long-term controlled clinical trial to show whether ATF helps to inhibit further periodontal breakdown in maintenance patients with high MSBI.

**Trial registration:**

This trial was registered in the WHO International Clinical Trials Registry Platform as DRKS00007672, date of registration: 21/01/2015.

**Electronic supplementary material:**

The online version of this article (doi:10.1186/s12903-016-0214-z) contains supplementary material, which is available to authorized users.

## Background

The link between oral health and general health is becoming generally accepted. Prevention of oral inflammatory diseases is considered to be a key element of good oral and systemic health [1]. Tools and chemical compounds in addition to mechanical plaque control may be helpful to support oral health. Additional chemical plaque control as part of domestic oral hygiene has always been playing an important role in the treatment of gingival inflammation [2, 3].

Patients with periodontitis need to be enrolled in a periodontal treatment protocol. During the initial visit, the patient’s medical and dental history is evaluated and clinical examinations and radiographic analyses are performed. The patients are being informed that periodontitis is an irreversible disease and that progress can be arrested by proper treatment. Periodontal treatment includes non-surgical and surgical procedures and domestic oral hygiene regimes. The maintenance phase after periodontal treatment is as important as the treatment itself and plays a decisive role whether the long-term outcome is successful or not [4]. Continuous domestic oral hygiene is indispensable to reach this aim and suitable antiseptics may help.

Chlorhexidine digluconate (CHX) is currently considered to be the most effective antiseptic mouthrinse due to its high substantivity and strong anti-bacterial effects and has been used in dentistry for many decades as liquid or gel [5]. Numerous studies have confirmed its efficacy in inhibition of plaque formation and reduction of gingival inflammation [6, 7]. CHX-containing mouthrinses have a significant antibacterial effect up to 7 h after its application [8]. Its efficacy is similar in mouthrinses with and without alcohol [9]. However, CHX has side effects such as reversible dysgeusia, black hairy tongue and tooth discolorations [5, 10–12] and is therefore not suitable for daily use in long-term periodontal maintenance. As a consequence, the search for effective alternatives without side effects continues.

Novel rinsing solutions should be tested for their application in periodontal maintenance for efficacy and side effects. Aluminium triformate (ATF), an aluminium salt, has been used for several decades in Europe to control “bleeding gums” and is considered to be safe for daily use. Other compounds of aluminium salts (aluminium-containing mouthrinses) have shown a reducing effect on bacterial growth and plaque formation [13, 14]. Application of alum (in combination with salt and vinegar) for mouth rinsing was advocated by Hippocrates approx. 2400 years ago [15]. Aluminium and other similar metals (polyvalent cations) are widely used in dental products [14]. ATF is available since 1967, but besides some clinical trials in the 80ies of last century that did not meet todays’ standards of good clinical practice, studies have not been performed so far to test its efficacy in the periodontal maintenance phase. ATF is likely anti-inflammatory due to its astringent characteristics, but data are not available for its effects on gingival inflammation and plaque formation. ATF creates a protective surface of denaturated keratins and other proteins as a colloidal layer through ionic bonds with proteins of the gingiva preventing penetration of bacterial compounds such as endotoxins into the underlying connective tissue.

The aim of this clinical short-term pilot study was to analyze the effects of ATF as active component in a commercially-available mouthrinse (Cional®; Kreussler & Co. GmbH, Wiesbaden, Germany) as an adjunct to mechanical oral hygiene in the maintenance phase of periodontal patients. The primary hypothesis was that the use of ATF leads to a stronger reduction of gingival inflammation than placebo. The secondary hypothesis was that the use of ATF reduces plaque formation and does not have significant side effects whereas the compliance of patients is good.

## Methods

### Experimental design

A pilot randomized placebo-controlled, 1-week double blind clinical trial with parallel groups was designed. The mouthrinse containing ATF is freely available without prescription at the pharmacy to be used for a period of maximally one week. We considered it necessary first to perform a short-term study as a pilot for an extensive clinical trial for two reasons: 1° we wanted to test the compliance of the patients who participated in the study and 2° we wanted to monitor side effects. Side effects such as allergic reaction or skin irritation have been reported as possible side effects. However, ATF-containing mouthrinse has been used for over 30 years and side effects have not been reported thus far.

### Study participants

Consecutive periodontal patients were screened at the Department of Operative Dentistry at the University Medical Center (Mainz, Germany) during August and September 2013. Patients who met the inclusion and exclusion criteria were recruited for enrollment.

The following patient inclusion criteria were applied:

- History of chronic periodontitis, having had active periodontal treatment including non-surgical and surgical therapies, and being in maintenance for ≥1 year;
- ≥18 years old;
- Willing to participate and willing to sign the informed consent;
- Having a modified sulcus bleeding index (MSBI) of ≥40 % [16];
- No pocket depths ≥6 mm;
- At least 20 teeth (at least 5 teeth in each quadrant);
- Systemically healthy.

Exclusion criteria:

- Known hypersensitivity to ATF;
- Known hypersensitivity to other ingredients of Cional® (Cremophor RH40, peppermint oil, mint oil, propylene glycol, glycerol);
- Any anti-inflammatory or antibiotic therapy < 3 months before treatment;
- Any other treatment during the study;
- Smoker;
- Pregnancy or wish for pregnancy;
- Lactating women;
- Participation in another clinical study;
- Lack of ability to participate in the screening appointments.

### Interventions

#### Screening visit

After a careful medical health evaluation, the oral health examination was carried out at the screening visit. The investigator (CE) controlled the inclusion/exclusion criteria and informed the patients about the details and aim of the study. The subjects signed the informed consent form when they accepted to participate voluntarily in the clinical trial.

#### Baseline visit

**Fig. 1:**
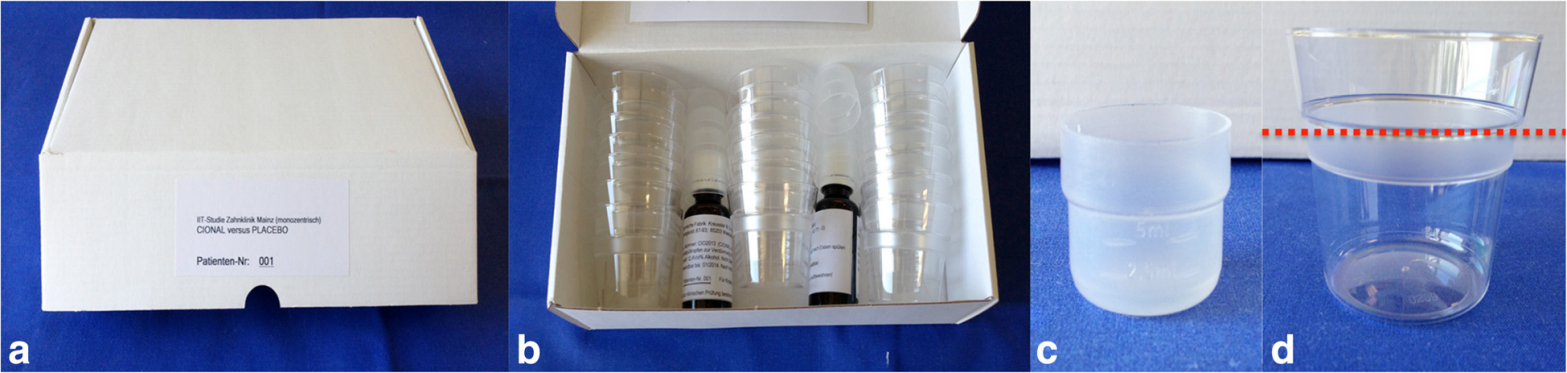
Preparation of the rinsing solution. Each participant received a box containing two bottles of the rinsing concentrate (**a** and **b**). Special cups of 2.5 ml (**c**) and 50 ml (**d**) were included in the box for the preparation of the rinsing solution. The special cup of 2.5 ml (**c**) was used to measure the mouth rinse concentrate that has to be diluted with water in the cup of 50 ml (**d**)

At the baseline visit, each participant was orally investigated (see below) and received an individual trial number. Following the recruitment, the patients were randomly allocated to either test or control group. Randomization was performed using random numbers from a computer-generated list provided by the manufacturer. Both, dentists and participants were masked to group allocation. All patients received their assigned product kit containing a non-labeled bottle (coded with numbers) of a concentrate that contained either a solution of ATF or the same solution but without ATF (placebo) and a special scaled container to dilute the rinsing solution with water. The patients were trained to mix the solution and each subject was given a written instruction (rinsing protocol) on how to use and dilute the mouthrinse. The rinsing solution consisted of 2.5 ml ATF or placebo from the masked bottle, which was to be diluted with water to 50 ml (Fig. 1). The participants were instructed to rinse 3 times daily for 30 s.

To check the compliance, the subjects had to make a note of the date, the time, the solution volume in milliliters and the rinsing time in seconds. The rinsing protocol served both to control the duration of the rinse and the motivation of the patient in terms of a memory aid. Furthermore, the subjects were asked to complete a questionnaire concerning the taste of the product, special sensation on tongue or mucosa and possible loss of taste sensation.

#### One-week reevaluation

After one week of rinsing, the used bottles as well as compliance forms were collected and the patients were orally investigated (see below) and asked for occurrence of any adverse effects.

### Clinical parameters

One calibrated examiner (CE), who was blinded for the content of the mouthrinse, performed all oral investigations. The gingival condition was evaluated during the baseline visit using MSBI (Additional file 1) and plaque was measured using the plaque index (PI) [17] and approximal plaque index (API) (Additional file 1) using plaque disclosing tablets (PD Produits Dentaires SA, Vevey, Switzerland) for 30 s and subsequent water rinsing. The clinical examinations were repeated after one week of rinsing with ATF or placebo mouthrinse (follow-up visit).

### Statistical analysis

As the current trial is a pilot study, no formal calculation on power and group sizes could be made a priori. Categorical parameters were described using absolute and relative frequencies, continuous parameters were described by mean, standard deviation and range. The age of the patients in the test and control group was analyzed for differences by the Student t-test. The gender distribution was analyzed by the Fisher’s exact test.

The primary endpoint of this study was MSBI. The baseline and MSBI follow-up levels were compared between groups using the Mann-Whitney test. The Wilcoxon signed-rank test within each group (baseline and follow-up visit) was used to assess the change in MSBI for ATF and placebo treatment. PI and API were analyzed in the same way.

Statistical analyses were performed using SPSS 22.0 (Chicago, IL, USA) and SAS 9.4 (Cary, NC, USA, 2002–2012) and the significance level was set at α = 0.05. Sample size calculation was performed using nQuery Advisor 5.0 (1995–2002, Janet Elashoff).

## Results

### Study population and patient characteristics

**Fig. 2:**
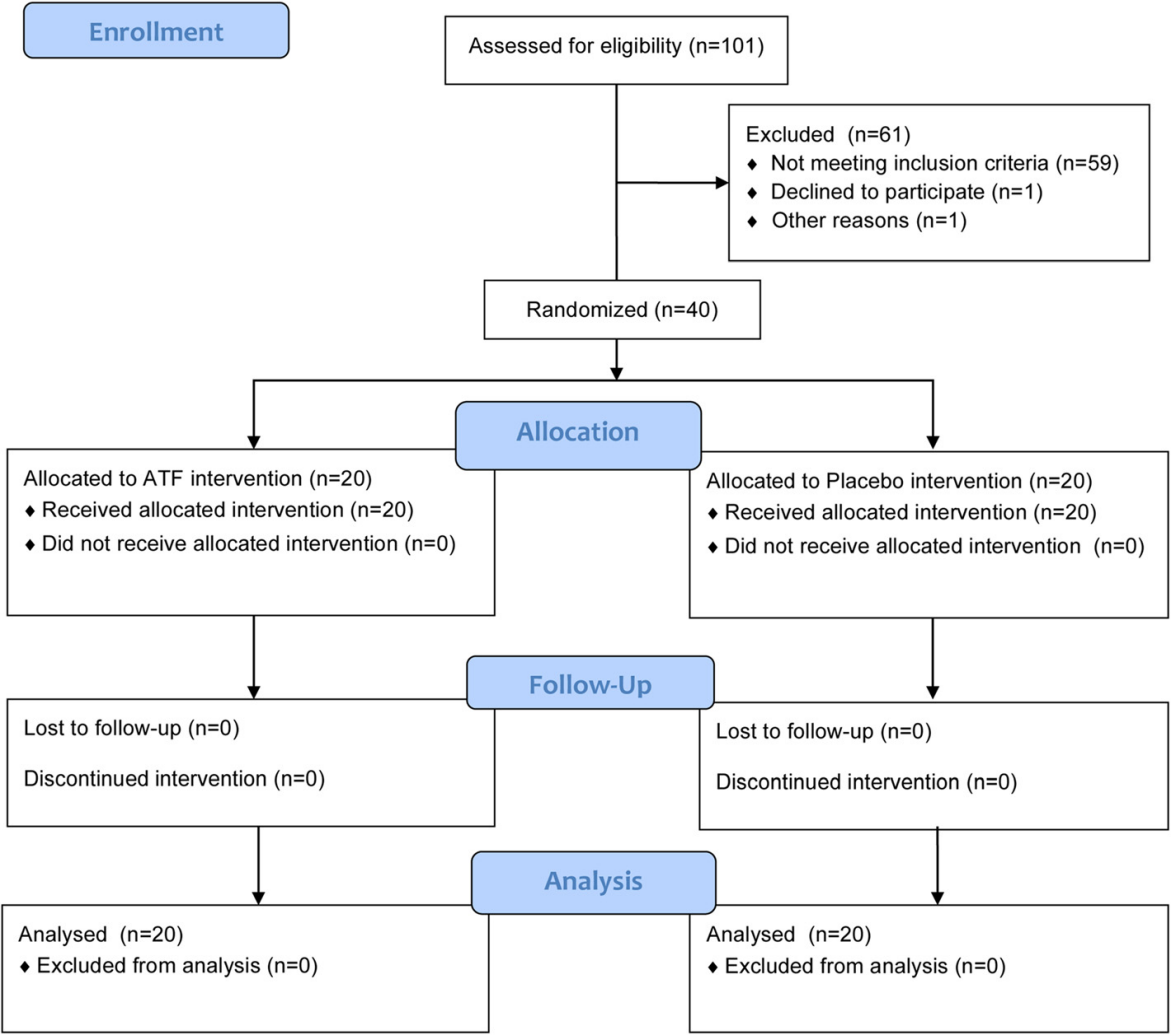
Study flow diagram according to CONSORT 2010

A total of 101 periodontal patients were assessed for eligibility and 42 met the inclusion and exclusion criteria and were enrolled (Fig. 2). One patient declined to participate and one patient withdrew. Thus, 40 patients were randomized and equally allocated to either the ATF group or the placebo group. All participants (*n* = 40) continued until the end of study and for all patients complete data sets were available (Fig. 2).

A total of 101 periodontal patients were assessed for eligibility. The age of participants ranged between 31 and 78 years and the majority of patients was female (65 % in the ATF group and 90 % in the placebo group). The mean age (60.6 and 58.6, respectively) did not differ between the ATF group and placebo group (Table 1). The number of teeth per group was comparable (25.9 teeth in the ATF group and 24.4 in the placebo group). The relative frequency of residual pockets was similar as well in the ATF group and placebo group (18.1 ± 12.3 and 17.1 ± 13.1 %, respectively; Table 1).

**Table 1:** Demographic data of the patients included in the study

Group	ATF (*n* = 20)	Placebo (*n* = 20)	*p*-Value
Age	60.6 ± 9.4	58.6 ± 10.1	0.636
Range	46–78	31–75	
Gender			
Female	13 (65 %)	18 (90 %)	0.058
Male	7 (35 %)	2 (10 %)	
Number of teeth	25.8 ± 2.9	24.3 ± 2.5	0.445
Number of sites	155.4 ± 18.5	146.7 ± 15.7	0.149
Range	120–186	129–174	
Number of residual pockets (4–5 mm)	28 ± 20.3	26.7 ± 21.1	0.440
Range	(1–86)	(9–93)	
Frequency of residual pockets (%)	18.1 ± 12.3	17.1 ± 13.1	0.398

Values represent descriptive means and standard deviations

### Clinical outcomes

The analysis of the data showed a notable improvement of the MSBI values in both groups after one week of rinsing (a reduction from 62.8 to 45.2 % for the ATF group and from 59.2 to 51.6 % for the placebo group at baseline and after 1 week rinsing, respectively; Table 2). The reduction in MSBI was significantly higher in the ATF group (*p* = 0.035).

**Table 2:** Clinical parameters at baseline and after one week rinsing (reevaluation)

Clinical parameter		ATF (*n* = 20)	Placebo (*n* = 20)	*p*-value (inter-group differences)
Primary outcome:				
MSBI	Baseline	62.8 ± 17.1	59.2 ± 14.8	0.529
	Reevaluation	45.2 ± 18.9	51.6 ± 19.5	0.383
	*p*-value (intra-group differences)	0.001	0.018	
	Reduction	17.6 ± 13.8	7.6 ± 11.3	0.035
Secondary outcome:				
PI	Baseline	0.7 ± 0.5	0.7 ± 0.4	0.640
	Reevaluation	0.8 ± 0.5	0.7 ± 0.4	0.445
	*p*-value (intra-group differences)	0.17	0.34	
	Reduction	0.09 ± 0.27	0.06 ± 0.26	0.947
API	Baseline	52.2 ± 19.1	43.3 ± 14.7	0.157
	Reevaluation	57.3 ± 20.8	45.1 ± 15.9	0.056
	*p*-value (intra-group differences)	0.177	0.602	
	Reduction	5.1 ± 14.9	1.8 ± 12.0	0.495

Values represent descriptive means and standard deviations for all indices

Oral hygiene and the amount of dental plaque were evaluated, analyzing PI and API. The differences of PI and API at baseline and after 1 week of rinsing were not significant (*p* = 0.947 for PI and *p* = 0.495 for API; Table 2).

### Compliance and subjective outcomes

All subjects (*n* = 40) returned the forms containing details about date, solution volume and rinsing time. All patients had rinsed during the entire week. Table 3 shows the subjective outcomes of the participants with respect to taste, oral sensations and personal assessment during the rinsing time. None of the participants complained about an unpleasant taste or negative or adverse effects (Table 3).

**Table 3:** Subjective outcomes

	ATF (*n* = 20)	Placebo (*n* = 20)
Taste		
Unpleasant	0	0
Pleasant	0	20
Reduction in taste sensation	0	0
Oral sensation		
Mouth dryness directly after rinsing	19	0
Roughness surface of the teeth, tongue and mucosa	20	0
Astringent feeling of the mucosa	18	0
Furry sensation of the oral cavity after rinsing	19	0
Metallic taste shortly after rinsing	19	0
Reduction of the gum bleeding during domestic oral hygiene procedures	19	6

Values represent absolute numbers of individuals with positive responses

On the other hand, patients did report on oral sensation. The majority of participants that had applied the ATF mouthrinse reported mouth dryness directly after rinsing, roughness of the surface of the teeth, tongue and mucosa, astringent feeling of the mucosa, furry sensation of the oral cavity after rinsing and metallic taste shortly after rinsing. Interestingly, 19 out of 20 participants in the ATF group reported a reduction of gum bleeding during domestic oral hygiene procedures (Table 3).

## Discussion

The present double blind placebo-controlled pilot trial is the first in vivo study that evaluates the efficacy of an ATF-containing mouthrinse used by periodontal patients in the maintenance phase. Fourty patients were enrolled and have completed this study. The primary hypothesis of this study was that ATF reduces the MSBI more than placebo without significant side effects has been confirmed.

After a period of 7 days, there was a notable reduction in gingival bleeding in both groups. The comparison between the two groups showed a significantly higher reduction in gingival bleeding as assessed by MSBI in the ATF group. This is consistent with patients’ subjective experience in the ATF group of reduced sulcus bleeding after rinsing. PI and API were not affected during this short-term application of an ATF-containing mouthrinse. It should be noted that the current rinsing period is relatively short, long-term effects of the rinsing with ATF need to be investigated. This pilot study was performed to demonstrate whether MSBI, PI and API were affected by an ATF-containing mouthrinse without side effects and whether the compliance of the patients included in the study was good.

This study was a pilot study and therefore not necessarily powered to detect significant differences between treatments. In this study, the estimated probability that ATF leads to a stronger decrease in MSBI (P(ΔMSBI_ATF_ < ΔMSBI_Placebo_)) was 0.3125. When calculating sample size and power under the assumption that P(ΔMSBI_ATF_ < ΔMSBI_Placebo_) = 0.3125 and significance level α = 0.05, 38 patients per group are needed to demonstrate this effect with 80 % power and 50 patients are needed to demonstrate this effect with 90 % power. With 20 patients per group, the calculated power is 53 %. Hence, further studies should include at least 100 patients in order to demonstrate efficacy of ATF.

The improvement of the MSBI values in the placebo group can be explained by the Hawthorne effect [18]. This phenomenon is considered to be based on increased attention, e.g. due to the participation in a clinical trial and the consciousness of being evaluated, leading to an improvement of productivity, or, in this case, a reduction in MSBI in the placebo group because of an improved oral hygiene during the study. However, a similar Hawthorne effect must be present in the ATF group, too. Because both patients and investigator were masked with respect to treatment, it is unlikely, that this effect differs between groups. The reduction in MSBI was significantly stronger in the ATF group than in the placebo group, which points at a true effect of ATF on MSBI.

Lang et al. [19] demonstrated the correlation between the amount of gingival bleeding and tooth survival in patients during periodontal maintenance. It was shown that periodontal patients with bleeding ≥16 % on probing had a higher risk of losing attachment than patients with <16 % bleeding on probing. After 4 consecutive maintenance appointments, patients with no bleeding on probing had a 20-fold lower risk of attachment loss than patients with bleeding on probing at the 4 maintenance appointments. These data indicate the importance of oral hygiene and control of gingival bleeding during the maintenance phase.

Ramberg et al. [20] showed a correlation between gingival inflammation and de novo plaque formation. Reduced gingival inflammation lowers the amount of crevicular fluid that contains proteins, which are metabolized by plaque microorganisms. As the quantity of crevicular fluid correlates with the grade of gingiva inflammation, a reduction of sulcus irritation decreases the inflammation and therefore prevents de novo plaque formation. Furthermore, Van der Velden [21] stated that gingival fluid is the major cause of de novo plaque formation. However, neither ATF rinse nor placebo rinse had any effect on the amount of dental plaque during the short experimental period of 7 days in our study. Putt et al. [14] showed a significant reduction in the amount of plaque after 2 and 4 weeks of rinsing and thus, it may well be that longer rinsing periods show effects on plaque levels as well.

With respect to the patients’ evaluation reports, ATF mouthrinse seems to be safe and not causing harmful side effects. Almost all patients in the ATF group reported the rough sensation of gingiva and teeth surface accompanied with a metallic taste. These sensations can be clearly explained by the astringent properties of aluminium solutions [22]. However, these sensations disappeared after a few minutes and not one patient indicated a desire to stop the rinsing, thus it is concluded that compliance of the patients included in the study is good.

The fast reduction in gingival bleeding may be useful as a pre-surgical measure in patients with inflamed gingiva. For protocols in which authors propose immediate periodontal surgery (without initial therapy) in patients with advanced periodontitis [23], it can be recommended that patients rinse with ATF during one week before surgery; the styptic effect of ATF may well be beneficial, but this application in untreated periodontitis patients is not yet tested and needs further investigation. ATF rinse may also be beneficial for patients with fixed orthodontic appliances during an acute period of gingival inflammation that are at risk for temporary periodontal destructive processes [24].

## Conclusions

ATF mouthrinse solution appears to be a promising adjunct to mechanical tools in the periodontal maintenance phase in our short-term pilot clinical trial. A long-term trial with a larger number of participants is needed to confirm our findings. The current study shows that the ATF rinse solution is well accepted by patients and does not have major and long-lasting side effects.

## Additional file

Additional file 1: Modified SBI and API (Lange, [16]). (DOCX 73 kb)

## Abbreviations

ATF: aluminium triformate
MSBI: modified sulcus bleeding index
API: approximal plaque index
PI: plaque index
