# Supplementary material for: Efficacy of an aluminium triformate mouthrinse during the maintenance phase in periodontal patients: a pilot double blind randomized placebo-controlled clinical trial
Source: BMC Oral Health. 2016 May 23;16:57. doi: 10.1186/s12903-016-0214-z (PMC4878033; doi:10.1186/s12903-016-0214-z)
Supplement: Additional file 1: — Modified SBI and API (Lange, [16]). (DOCX 73 kb) [file 12903_2016_214_MOESM1_ESM.docx]

**Additional file 1**

**Modified SBI** (Lange, 1986)

This dichotomous index (yes/no-decision) regards the bleeding in the interdental spaces after probing with a periodontal probe. In the first quadrant, the spaces are checked from the lingual/palatal side, in the second quadrant from buccal side. The third quadrant is probed from lingual, the fourth from buccal side again.

In total, a maximum of 28 measuring sites can be achieved.

The index is calculated as follows:

∑ positive scored sites

∑ all available sites

>50% strong and generalized periodontal inflammation

>21-50% moderate inflammation

10-20% mild inflammation

<10 % clinically normal periodontium

**API** (Lange, 1986)

Similar to the modified SBI, this dichotomous regards the interdental spaces alternating from the buccal or the lingual/palatal surfaces as described above. It evaluates interdental plaque accumulation. For this purpose, plaque is stained with plaque disclosing tablets and thereafter the (maximum 28) interdental spaces are scored quadrant-wise.

The index is calculated as follows:

∑ positive scored sites

∑ all available sites

>70% insufficient oral hygiene

70-40% moderate oral hygiene

25-39% acceptable oral hygiene

<25% optimal oral hygiene
